# Protein-Calorie Malnutrition Is Associated with Altered Colonic Mucosal Microbiota in Inflammatory Bowel Disease

**DOI:** 10.3390/nu17233775

**Published:** 2025-12-01

**Authors:** Hyo-Joon Yang, Melissa Corson, Ezinne Aja, Ellen Spartz, Berkeley N. Limketkai, Jonathan P. Jacobs

**Affiliations:** 1 Division of Gastroenterology, Department of Internal Medicine and Gastrointestinal Cancer Center, Kangbuk Samsung Hospital, Sungkyunkwan University School of Medicine, Seoul 03181, Republic of Korea; hyojoonyang@gmail.com; 2 Vatche and Tamar Manoukian Division of Digestive Diseases, Department of Medicine, David Geffen School of Medicine at UCLA, Los Angeles, CA 90095, USAeaja@mednet.ucla.edu (E.A.); espartz@mednet.ucla.edu (E.S.); 3 Goodman-Luskin Microbiome Center, David Geffen School of Medicine at UCLA, Los Angeles, CA 90095, USA; 4 Division of Gastroenterology, Hepatology and Parenteral Nutrition, Veterans Administration Greater Los Angeles Healthcare System, Los Angeles, CA 90073, USA

**Keywords:** inflammatory bowel disease, protein-calorie malnutrition, colonic microbiota, *Bifidobacterium*

## Abstract

**Background/Objectives:** Protein-calorie malnutrition (PCM) is common among patients with inflammatory bowel disease (IBD). However, the relationship between PCM and the gut microbiota in patients with IBD remains unclear. This study aimed to investigate the association between PCM and the colonic mucosal microbiota in patients with IBD. **Methods:** Colonic mucosal samples were obtained from 24 IBD patients with PCM and 24 IBD type-matched patients without PCM. PCM was defined as a body mass index (BMI) < 18.5 kg/m^2^ and/or weight loss of ≥10% within the preceding 6 months. The full-length bacterial 16S ribosomal RNA gene (V1–V9) was sequenced using the PacBio Sequel IIe. Alpha and beta diversity and species-level differential abundance were analyzed, adjusting for age, sex, BMI, and disease type. **Results:** Among 48 patients (36 Crohn’s disease and 12 ulcerative colitis), diversity indices (Chao1, *p* = 0.474; Shannon, *p* = 0.931) and overall composition (Bray–Curtis, *p* = 0.719) did not differ by PCM status, although microbial composition was associated with age (*p* = 0.011) and biopsy-site inflammation (*p* = 0.001). PCM was associated with 12 differentially abundant taxa, including enrichment of *Intestinibacter bartlettii* and depletion of *Bifidobacterium longum*, *Sphingomonas leidyi*, and *Clostridium innocuum*, along with changes in several previously unclassified species. **Conclusions:** IBD patients with PCM exhibited shifts in the colonic mucosal microbiota including reduction in *Bifidobacterium longum*, a well-known probiotic. Further investigations into the role of the microbiota in PCM in IBD patients and the potential beneficial effects of probiotics are warranted.

## 1. Introduction

Protein-calorie malnutrition (PCM) is common among patients with inflammatory bowel disease (IBD). In a previous nationwide study, the prevalence of PCM was 11.6% and 12.9% among hospitalized patients with Crohn’s disease (CD) and ulcerative colitis (UC), respectively, compared to 1.9% in non-IBD patients in the Unites States [1]. The pathogenesis of malnutrition in patients with IBD is multifactorial. Chronic mucosal inflammation plays an important role, as it impairs nutrient absorption, increases intestinal protein loss, and disrupts epithelial integrity. This ongoing mucosal inflammation stimulates the release of inflammatory mediators such as tumor necrosis factor-α, interleukin-6, and leptin, which drive systemic inflammation, anorexia, hypermetabolism, and muscle catabolism, thereby further exacerbating malnutrition [1,2]. Additional contributing factors include medication effects, fasting for medical procedures, and prolonged restrictive diets that further limit nutrient intake [3,4]. PCM has been associated with increased hospitalization, reduced therapeutic response, and impaired quality of life in patients with IBD [5,6]. Clinical guidelines recommend screening of nutritional status and early nutritional support for IBD patients with malnutrition [7,8].

Malnutrition contributes to intestinal edema, barrier dysfunction, and reduced wound healing [2,9]. It also compromises both local and systemic immune responses through diminished cell-mediated immunity, complement activity, and phagocyte function, thereby increasing susceptibility to infection [10]. The gut microbiota represents a possible mediator between IBD and malnutrition. Substantial dysbiosis of the gut microbiota has been reported in patients with IBD compared with healthy individuals even with clinical and endoscopic remission [11,12,13]. Malnutrition per se is associated with an immature gut microbiota enriched in facultative anaerobes, Gram-negative bacteria, and potential pathogens [14]. Childhood malnutrition is associated with an immature and disrupted gut microbiota. Importantly, this microbiota immaturity is not fully restored by nutritional therapy alone, indicating that microbiota-directed interventions may be required to achieve sustained recovery [15]. However, the specific relationship between PCM and the gut microbiota in patients with IBD remains insufficiently characterized. Therefore, this study aimed to investigate the association between PCM and the colonic mucosal microbiota in patients with IBD.

## 2. Materials and Methods

### 2.1. Cohort Recruitment and Sample Collection

For this study, we recruited patients with IBD and concurrent PCM and disease type-matched IBD patients without PCM who were undergoing colonoscopy between 16 November 2012 and 13 November 2014. The inclusion criteria were age of 18 years or older and the diagnosis of IBD. PCM was defined as a body mass index (BMI) < 18.5 kg/m^2^ and/or weight loss of ≥10% within the preceding 6 months. Patients were excluded if they had used antibiotics in the past month.

We collected clinical data including age, sex, BMI, type of disease (CD or UC), disease duration, extent of disease, and medication use. During colonoscopy, colonic mucosal samples were obtained using biopsy forceps, immediately frozen at −20 °C, and stored at −80 °C until future microbiome analysis. Sampled regions included the rectum (*n* = 1), sigmoid colon (*n* = 29), descending colon (*n* = 5), transverse colon (*n* = 5), ascending colon (*n* = 3), cecum (*n* = 4), and colon pouch (*n* = 1). We also recorded whether the samples were from mucosa with endoscopic inflammation or from normal-appearing mucosa without inflammation. The study was approved by the Institutional Review Board of University of California Los Angeles (IRB No. 12-000420). Written informed consent was obtained from all the patients prior to their participation in this study.

### 2.2. Full-Length 16S rRNA Gene Sequencing

Genomic DNA was extracted from colonic mucosal biopsy samples using the ZymoBIOMICS DNA/RNA Miniprep kit (ZYMO Research Corp., Irvine, CA, USA), following the manufacturer’s instructions. The full-length bacterial 16S rRNA gene (V1–V9 region) was amplified with polymerase chain reaction (PCR) using KAPA HiFi HotStart Taq polymerase (KAPA Biosystems, Boston, MA, USA) and barcoded forward and reverse primers designed by Pacific Biosciences (PacBio, Menlo Park, CA, USA). Amplicons were pooled, and 1 μg of pooled DNA was used for purification and preparation of multiplexed amplicon libraries using the PacBio SMRTbell prep kit 3.0 (PacBio). Long-read sequencing was conducted using a PacBio Sequel IIe (PacBio). Initial processing of the circular consensus sequencing data was carried out with SMRT Link to produce high-quality long reads. Further processing was performed in DADA2 to identify full-length amplicon sequence variants (ASVs), including primer sequence removal; filtering of low-quality sequences, short (<1000 bp) or long (>1600 bp) sequences, and sequences with wrong base; denoising; and chimera removal [16]. Taxonomy was assigned using the RDP classifier implemented in the assignTaxonomy function of the R package dada2 v1.18.0 and the Silva v138 database [17]. The sequencing depth of the samples after processing ranged from 2430 to 20,949, with a mean depth of 8105.

### 2.3. Microbial Profiling

Microbial alpha diversity was evaluated using the Chao1 index to estimate species richness and the Shannon index to assess both richness and evenness, employing the estimate_richness function of Phyloseq v1.34.0 in R (version 4.0.0; R Foundation for Statistical Computing, Vienna, Austria) [18]. Alpha diversity analyses used sequence data rarefied to a depth 2430 reads [19]. The metrics were compared between PCM and control using the Mann–Whitney U test. To account for potential confounding variables while considering the small sample size of the cohort, the associations between the alpha diversity metrics and PCM were adjusted in two stages. Initially, adjustments were made for age, sex, BMI, and disease type (CD vs. UC) using mixed effects multivariate analysis of variance (MANOVA) models. Subsequently, further adjustments were applied in the models for disease duration, disease location (colonic vs. extracolonic only), immunomodulator/steroid use, biologics use, and the presence of inflammation at the sample biopsy site. Missing covariate values were imputed using the k-nearest neighbor (kNN) algorithm.

Beta diversity was assessed at the species level using non-rarefied sequence data, after filtering out taxa with fewer than three non-zero counts. Bray–Curtis dissimilarity was calculated with the vegdist function from the R package vegan v2.6-4 [14]. Principal coordinates analysis (PCoA) was performed using the pcoa function in the R package ape v5.7.1 to visualize these results. Data ellipses capturing 90% of the data points in the PCoA plot were added using the stat_ellipse function from ggplot2 v3.4.4 in R. The statistical significance of microbial compositional differences associated with PCM was assessed using permutational multivariate analysis of variance (PERMANOVA) of Bray–Curtis dissimilarity, utilizing the adonis function in vegan v2.6-4 with 10,000 permutations [20]. The difference was adjusted for age, sex, BMI, and disease type through repeated measures PERMANOVA with 10,000 permutations [21]. Subsequently, additional adjustments were made for disease duration, disease location, immunomodulator/steroid use, biologics use, and inflammation at the biopsy site.

Differentially abundant taxa at the species level between PCM and control were analyzed using non-rarefied sequence data following the removal of taxa with fewer than three non-zero counts. This analysis was conducted with DESeq2 v1.30.1 integrated in Phyloseq v1.34.0 in R. DESeq2 normalizes sequencing data using size factors to allow for sequencing depth differences among samples, applies negative binomial models to account for over-dispersion, and employs adaptive shrinkage to improve stability with small sample sizes [22]. The differences between PCM and control were adjusted for age, sex, BMI, disease type, disease duration, disease location, immunomodulatory/steroid use, biologics use, and inflammation at the biopsy site. To account for multiple comparisons, the differential abundance results were adjusted using the qvalue function v2.22.0 in R v4.0.0, and a *q*-value < 0.1 was considered significant.

### 2.4. Subgroup Analysis

We conducted subgroup analyses to further examine microbiome differences associated with PCM. First, in patients with CD, we compared alpha diversity, beta diversity, and taxonomic differences between PCM and control, adjusting for all covariates described above except for disease type, which was not applicable in this analysis. We additionally performed two separate subgroup analyses: one restricted to patients without a history of bowel surgery and another restricted to those with such history. In these subgroups, analyses were adjusted for all covariates.

## 3. Results

In this study, we collected colonic mucosal samples from 24 IBD patients with concurrent PCM and 24 IBD type-matched patients without PCM during the study period. There were 18 pairs of patients with CD and 6 pairs of patients with UC. Patient demographics and clinical characteristics are summarized in Table 1. The median BMI in patients with PCM was 18.2 kg/m^2^ (interquartile range [IQR], 17.2–22.6), which was significantly lower than that of controls (median, 24.9 kg/m^2^; IQR, 21.3–27.0; *p* = 0.001). In addition, patients with PCM were significantly younger (*p* = 0.007), more likely to be female (*p* = 0.009), and had shorter disease duration (*p* = 0.038) compared to controls. In both groups, more than half of the patients were receiving either immunomodulators or corticosteroids, and more than half were also treated with biologic agents. In addition, 41.7% of patients with PCM and 50% of those without PCM had a history of bowel surgery.

### 3.1. Microbial Diversity and Overall Composition of the Colonic Mucosal Microbiota Were Not Significantly Altered in IBD Patients with PCM Compared to Those Without PCM

**Figure 1:**
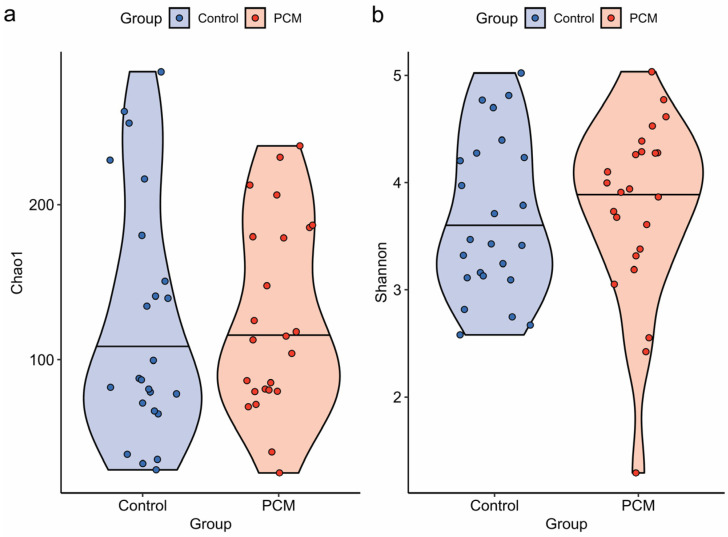
Protein-calorie malnutrition (PCM) was not significantly associated with differences in microbial alpha diversity in inflammatory bowel disease patients. Alpha diversity metrics, including (**a**) Chao1 and (**b**) Shannon indices, were compared between PCM and control. Multivariate analysis of variance, adjusting for age, sex, body mass index, disease type (Crohn’s disease vs. ulcerative colitis), disease duration, disease location (colonic vs. extracolonic only), immunomodulator/steroid use, biologics use, and the presence of inflammation at the sample biopsy site, revealed no significant differences in these indices between PCM and control both before adjustment (*p* = 0.658 and *p* = 0.431, respectively) and after adjustment (*p* = 0.474 and *p* = 0.931, respectively).

Bacterial diversity in the colonic mucosal microbiota of IBD patients with PCM was not significantly different compared to those with IBD without PCM by Chao1 index (microbial richness alone) (*p* = 0.658) and Shannon index (combined richness and evenness) (*p* = 0.431) (Figure 1). Similar results were obtained after adjusting for age, sex, BMI, and disease type (*p* = 0.955 and *p* = 0.630 for Chao1 and Shannon indices, respectively). Similar results were obtained after further adjustment for disease duration, disease location, immunomodulatory/steroid use, biologics use, and inflammation at the biopsy site (*p* = 0.474 and *p* = 0.931 for Chao1 and Shannon indices, respectively).

**Figure 2:**
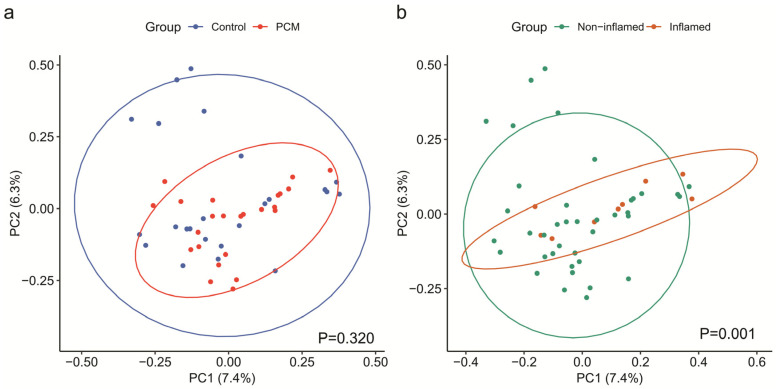
Biopsy site inflammation but not protein-calorie malnutrition (PCM) was significantly associated with microbial beta diversity in inflammatory bowel disease patients. (**a**) Beta diversity, as estimated by Bray–Curtis dissimilarity, was visualized using principal coordinates analysis (PCoA). Significance of differences between PCM and control was determined using repeated measures permutational multivariate analysis of variance with 10,000 permutations, adjusting for age, sex, body mass index, disease type (Crohn’s disease vs. ulcerative colitis), disease duration, disease location (colonic vs. extracolonic only), immunomodulator/steroid use, biologics use, and the presence of the sample biopsy site. Beta diversity did not significantly differ between PCM and control before adjustment (*p* = 0.320) and after adjustment (*p* = 0.719). (**b**) In contrast, beta diversity differed significantly according to the presence of inflammation at the biopsy site after adjustment for covariates (*p* = 0.001).

Colonic mucosal microbial community composition was then compared between PCM and control (beta diversity) by Bray–Curtis dissimilarity and visualized by PCoA (Figure 2). Microbial composition did not significantly differ between PCM and control in an unadjusted PERMANOVA analysis (*p* = 0.320). We conducted repeated measure PERMANOVA to compare microbial composition after adjusting for covariates. However, there was still no significant difference between PCM and controls after the initial adjustment (*p* = 0.644) or after further adjustment (*p* = 0.719). In contrast, microbial composition was significantly associated with age (*p* = 0.011) and presence of inflammation at the biopsy site (*p* = 0.001), demonstrating that our analysis detected expected relationships with the microbiome.

### 3.2. Colonic Mucosal Microbiota of IBD Patients with PCM Were Characterized by Species-Level Differences in Taxonomic Abundance

**Figure 3:**
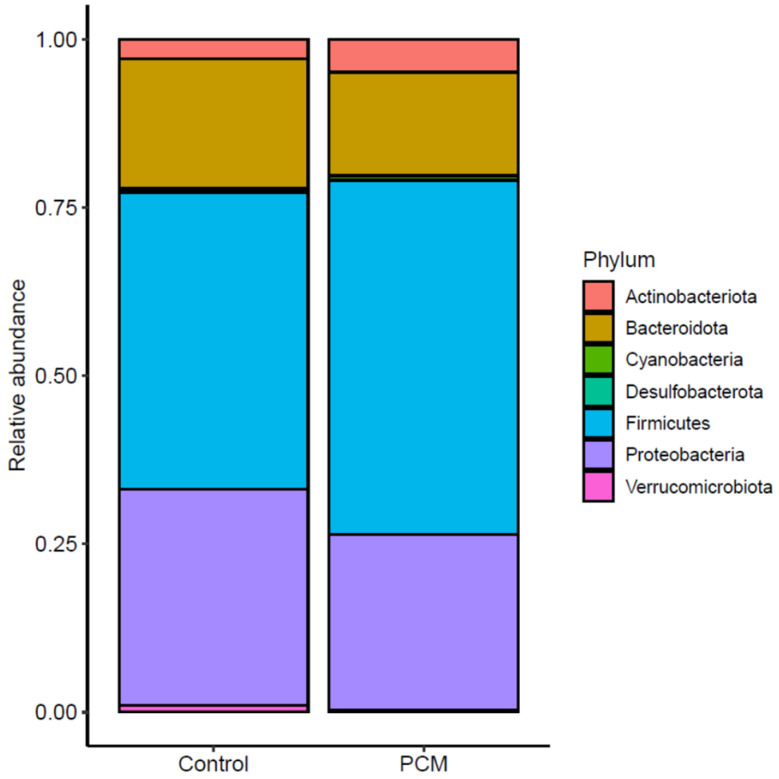
Most abundant phyla were similar between protein-calorie malnutrition (PCM) and control groups. Taxonomic composition at the phylum level was compared between PCM and control groups. The relative abundances of the major phyla—Firmicutes, Proteobacteria, and Bacteroidota—did not differ significantly different between groups. Desulfobacterota was significantly reduced in PCM compared to control (*q* < 0.1), although it represented only a minor portion of the colonic mucosal microbiota.

**Figure 4:**
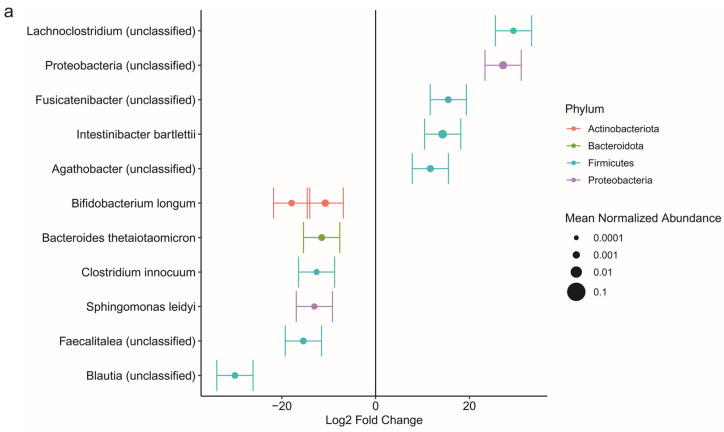

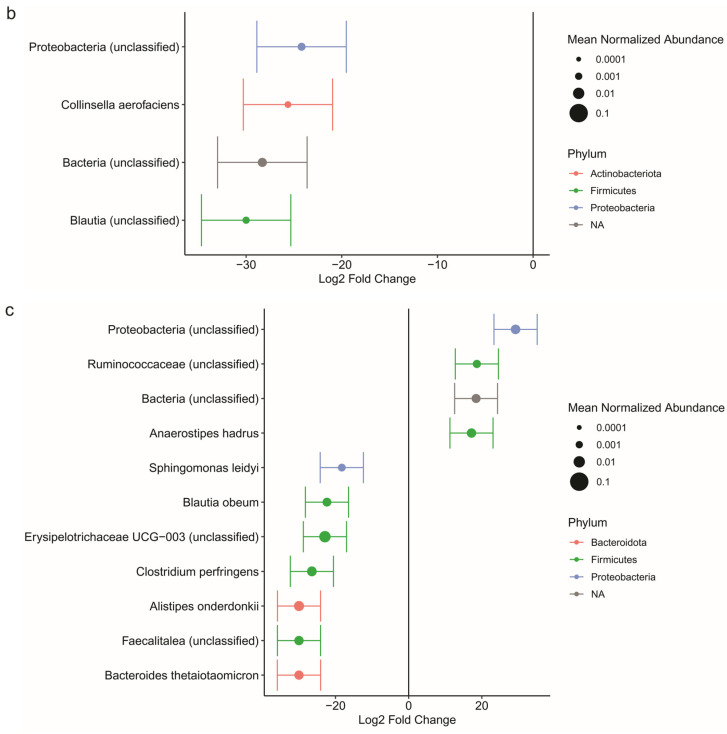
The colonic mucosal microbiota differed significantly at the species level between inflammatory bowel disease patients with and without protein–calorie malnutrition (PCM). (**a**) Differentially abundant amplicon sequence variants (ASVs; roughly corresponding to species) identified by DESeq2 analysis are shown with effect sizes represented as log_2_ fold change between PCM and control. Bars indicate the standard error of the effect size estimate. The size of each dot in the figure corresponds to normalized relative abundance, and color denotes phylum of the ASV. Unclassified ASVs at the species level are labeled as unclassified members of their respective genera. Significance was defined as *q* < 0.1 after adjusting for age, sex, body mass index, disease type (Crohn’s disease vs. ulcerative colitis), disease duration, disease location (colonic vs. extracolonic only), immunomodulator/steroid use, biologics use, and the presence of inflammation at the sample biopsy site. (**b**) Differentially abundant ASVs in the subgroup analysis of patients with Crohn’s disease. The results were adjusted for age, sex, body mass index, disease duration, disease location, immunomodulator/steroid use, biologics use, and the presence of inflammation at the sample biopsy site. (**c**) Differentially abundant ASVs in the subgroup of patients without a history of bowel surgery, adjusting for all covariates.

We evaluated taxonomic alterations associated with PCM using the DESeq2 method, which applies an empirical Bayesian approach to shrink dispersion and fits the data to negative binomial models. At the phylum level, Firmicutes, Proteobacteria, and Bacteroidota were the most abundant in both groups (Figure 3). The only differentially abundant phylum between PCM and control was Desulfobacterota (*q* = 0.036), which was reduced in PCM compared to control, despite representing only a minor portion of the colonic mucosal microbiota. At the species level, however, we identified 12 differentially abundant ASVs between PCM and control (Figure 4a). PCM was associated with increased abundance of unclassified *Lachnoclostridium*, unclassified Proteobacteria, unclassified *Fusicatenibacter*, *Intestinibacter bartlettii*, and unclassified *Agathobacter* compared to control. PCM was also associated with decreased abundance of unclassified *Blautia*, two ASVs belonging to *Bifidobacterium longum*, unclassified *Faecalitalea*, *Sphingomonas leidyi*, *Clostridium innocuum*, and *Bacteroides thetaiomicron*.

### 3.3. Subgroup Analysis Revealed That Unclassified Blautia Was Significantly Decreased in Both the Overall Cohort and CD Subgroup

We performed subgroup analysis in patients with CD with and without PCM. Subgroup analysis was not performed for UC due to small sample size. In the adjusted analysis for age, sex, BMI, disease duration, disease location, immunomodulator/steroid use, biologics use, and biopsy site inflammation, there was no significant difference in Chao1 and Shannon indices between CD with PCM and controls (*p* = 0.472 and *p* = 0.887, respectively). Microbial community composition was not significantly different between CD with PCM and control in the adjusted analysis (*p* = 0.379). However, the composition was significantly different according to age (*p* = 0.035) and biopsy site inflammation (*p* = 0.044). In the DESeq2 analysis, four taxa were significantly depleted in CD with PCM compared to control: unclassified *Blautia*, unclassified Bacteria, *Collinsella aerofaciens*, and unclassified Proteobacteria (Figure 4b). The significant decrease in the relative abundance of unclassified *Blautia* in patients with PCM compared to control was consistent across both the overall cohort and the CD subgroup.

In the subgroup of patients without a history of bowel surgery, alpha diversity (Chao1, *p* = 0.470; Shannon, *p* = 0.219) and beta diversity (*p* = 0.462) did not significantly differ between PCM and controls. DESeq2 analysis identified 11 differentially abundant taxa, and among them the depletion of *Bacteroides thetaiotaomicron* and *Sphingomonas leidyi* associated with PCM was consistent with the findings in the overall cohort (Figure 4c). In the subgroup of patients with a history of bowel surgery, alpha diversity (Chao1, *p* = 0.370; Shannon, *p* = 0.138) and beta diversity (*p* = 0.369) also did not significantly differ between PCM and controls. Furthermore, DESeq2 analysis did not identify any differentially abundant taxa between the two groups.

## 4. Discussion

In this study, we compared the colonic mucosal microbiota between IBD patients with and without PCM using full-length 16S rRNA gene sequencing. We found no significant differences in alpha or beta diversity according to the presence of PCM. However, taxonomic analysis revealed 12 differentially abundant species between IBD patients with and without PCM, including the depletion of two ASVs belonging to *Bifidobacterium longum*, a well-known probiotic. These findings raise the possibility that alterations in the colonic microbiota may contribute to PCM in patients with IBD.

The gut microbiota is increasingly recognized as a driver of mucosal inflammation in the pathogenesis of IBD. Patients with IBD exhibit reduced microbial diversity and depletion of beneficial bacteria, including short-chain fatty acid (SCFA)-producing taxa [11,21]. SCFAs are essential for maintaining epithelial integrity and immune homeostasis in the colonic mucosa [12]. Similarly, malnutrition is characterized by distinct microbial alterations with an enrichment of potential pathogens, which promote lipopolysaccharide release, endotoxemia, and both mucosal and systemic inflammation [14]. Despite these well-established associations between the microbiota and each condition individually, the microbial characteristics of IBD patients complicated by PCM have remained largely unexplored. Our findings address this gap by demonstrating that PCM in IBD is not accompanied by global diversity changes but rather by specific taxonomic differences, suggesting selective microbial adaptations that may link nutritional status with mucosal inflammation in IBD.

Among the taxa showing differential abundance, *Bifidobacterium longum* was of particular interest. Notably, two ASVs belonging to *Bifidobacterium longum* were significantly depleted in IBD patients with PCM. *Bifidobacterium longum* is a well-known probiotic that has been shown in in vitro and animal studies to attenuate intestinal inflammation by downregulating inflammatory cytokines such as TNF-α, IL-1β, IL-6, and IL-8, and by inhibiting the NF-κB and MAPK signaling pathways [23]. In experimental colitis models, *Bifidobacterium longum* supplementation reduced the abundance of pathogenic bacteria and mitigated mucosal injury [24,25]. In the management of malnutrition, however, dietary intervention remains the cornerstone of therapy [7,8,26]. High intake of vegetables and restriction of animal fat and protein have been associated with a more protective gut microbial composition and a lower risk of relapse in CD [27,28]. Although several studies have reported beneficial effects of probiotics in reducing IBD relapse, clinical data specifically examining *Bifidobacterium longum* supplementation are scarce [29,30]. Nonetheless, a recent randomized controlled trial demonstrated that a probiotic supplement containing *Bifidobacterium* and *Lactobacillus* increased body weight, BMI, body fat and muscle mass, as well as the intake of micronutrients and fiber in patients with CD [31]. Taken together with these findings, our results suggest that probiotics such as *Bifidobacterium longum* may provide additional benefit by supporting both inflammatory control and nutritional recovery in IBD-associated PCM. Further research is warranted to elucidate whether *Bifidobacterium longum* administration can ameliorate malnutrition and mucosal inflammation in this patient population.

Other taxonomic changes observed in our study further support that microbial adaptation to malnutrition has occurred. Among the taxa that were reduced in IBD patients with PCM, *Bacteroides thetaiotaomicron*, a known producer of SCFAs, showed decreased abundance, which is consistent with previous findings that this species exhibits reduced viability under glucose-depleted conditions [32]. In contrast, *Intestinibacter bartlettii* was increased, which may reflect reduced intestinal gluconeogenesis. A previous study suggested that metformin-induced alterations in the gut microbiota, including reduced *Intestinibacter* abundance, may enhance intestinal gluconeogenesis in patients with type 2 diabetes mellitus [33]. Furthermore, *Collinsella aerofaciens*, which was reduced in the CD subgroup, has been reported to be depleted in IBD, particularly in CD and in individuals with low BMI [34,35,36]. Collectively, these findings suggest that IBD patients with PCM exhibit an aggravation of dysbiosis involving both inflammatory and metabolic pathways. However, the observed reduction in Clostridium *innocuum*, an opportunistic pathogen previously associated with infection in IBD patients [37], and the decrease in Sphingomonas *leidyi*, a species with no known link to IBD or host metabolism, are not readily explained by existing evidence.

Our study, which employed full-length 16S rRNA gene sequencing, enabled the identification of previously unclassified species that may have been overlooked in conventional short-read analyses [38]. Among these, unknown species belonging to the genera *Blautia* and *Faecalitalea* were decreased in IBD patients with PCM. This finding is consistent with prior reports showing that *Blautia*, a known SCFA-producing genus, is reduced in IBD [39], and that *Faecalitalea*, a butyrate producer, has been implicated in improving insulin resistance in diabetes [40]. Conversely, an unclassified *Lachnoclostridium* species was increased; notably, *Lachnoclostridium* has been reported to be enriched in CD, particularly in fibrostenotic phenotypes [41,42]. In contrast, we observed an increase in unclassified species of *Fusicatenibacter* and *Agathobacter*, although other species within these genera, such as *Fusicatenibacter saccharivorans* and *Agathobacter rectalis*, were reduced in active UC and IBD, respectively [43,44]. These findings warrant further taxonomic and functional characterization to clarify their biological significance. Taken together, our results suggest that IBD patients with PCM harbor distinct alterations even among previously unclassified taxa, highlighting potential links between nutritional status, inflammation, and microbial metabolism that merit further investigation.

It is noteworthy that approximately half of patients in our cohort had a history of bowel surgery, which is common among individuals with CD. Intestinal resection in CD has been associated with decreased alpha diversity, altered beta diversity, increased Proteobacteria, and reductions in Bacteroidetes and Firmicutes [45]. Thus, if one group had a higher proportion of patients with prior intestinal surgery, differences in diversity metrics could have been biased toward that group. In our study, however, the proportions of surgical history were comparable between the PCM and control groups, reducing the likelihood of such confounding. In addition, a subgroup analysis restricted to patients with prior surgery did not yield significant results, likely due to the small sample size. Taken together, these findings suggest that the inclusion of patients with surgical history is unlikely to have introduced meaningful bias into our analysis.

This study has several limitations. First, the sample size was relatively small, which limits the generalizability of our findings. However, given the paucity of microbiome studies focusing on PCM in IBD, even a modest cohort provides meaningful insights into this underexplored area. Second, detailed dietary information was not available, which may have influenced both the presence of PCM and the gut microbial composition. Future studies incorporating dietary assessment and larger, multi-center cohorts are warranted to validate and extend our observations.

## 5. Conclusions

IBD patients with PCM exhibited distinct alterations in the colonic mucosal microbiota, including a reduction in *Bifidobacterium longum*, a well-known probiotic species with anti-inflammatory and gut barrier-enhancing properties. These findings suggest that PCM may exacerbate intestinal dysbiosis and inflammation in IBD and highlight the need for further investigation into potential synergistic interactions between nutritional status, microbial imbalance, and disease activity. Moreover, future studies should explore whether probiotic supplementation, particularly with *Bifidobacterium longum*, could help restore microbial and nutritional homeostasis in IBD-associated PCM.

## Figures and Tables

**Table 1:** Demographics of patients included in the study.

	PCM (*n* = 24)	Control (*n* = 24)	*p*-Value
Age (years)	32.5 (27.25–46.75)	55 (34–63)	0.007
Gender			0.009
Male	6 (25.0)	15 (62.5)	
Female	18 (75.0)	9 (37.5)	
Body mass index (kg/m^2^)	18.2 (17.2–22.6)	24.9 (21.3–27.0)	0.001
Disease type			1.000
Crohn’s disease	18 (75.0)	18 (75.0)	
Ulcerative colitis	6 (25.0)	6 (25.0)	
Duration (years)	9 (2–14.25)	13.5 (7.25–34)	0.038
Location			1.000
Colonic	20 (83.3)	19 (79.2)	
Extracolonic only	4 (16.7)	5 (20.8)	
Medication			
Immunomodulator/Steroid	12 (50.0)	13 (54.2)	0.773
Biologic	14 (58.3)	15 (62.5)	0.768
Biopsy site inflammation			1.000
Non-inflamed	19 (79.2)	20 (83.3)	
Inflamed	5 (20.8)	4 (16.7)	
Disease activity			0.311
Active	10 (41.7)	10 (41.7)	
Remission	6 (25.0)	10 (41.7)	
Unknown	8 (33.3)	4 (16.7)	
Montreal classification			
Crohn’s disease, L1/L2/L3/+L4/NA	4/5/8/1/1	5/2/9/4/2	
Crohn’s disease, B1/B2/B3/+p/NA	6/3/8/5/1	3/5/4/5/6	
Ulcerative colitis, E1/E2/E3/NA	1/3/1/1	0/1/4/1	
History of bowel surgery			0.562
Yes	10 (41.7)	12 (50.0)	
No	13 (54.2)	10 (41.7)	
Unknown	1 (4.2)	2 (8.3)	

Continuous variables are shown as median (interquartile range), and categorical variables are shown as number (percentage). Significance of demographic data was determined by Chi-square test or Fisher’s exact test for categorical data and the Wilcoxon rank-sum test for continuous data. PCM, protein-calorie malnutrition; NA, not available.

## Data Availability

Raw sequencing data and associated metadata are publicly accessible through NCBI Bioproject PRJNA1348349.

## Abbreviations

The following abbreviations are used in this manuscript:

PCM	Protein-calorie malnutrition
IBD	Inflammatory bowel disease
BMI	Body mass index
CD	Crohn’s disease
UC	Ulcerative colitis
PCR	Polymerase chain reaction
ASV	Amplicon sequence variant
MANOVA	Multivariate analysis of variance
kNN	k-nearest neighbor
PCoA	Principal coordinates analysis
PERMANOVA	Permutational multivariate analysis of variance
IQR	Interquartile range
SCFA	Short-chain fatty acid
